# MRI-Based Prediction of Macrovascular Causes of Intracerebral Hemorrhage: The MACRO Score

**DOI:** 10.1212/WNL.0000000000209950

**Published:** 2024-10-31

**Authors:** Simon Fandler-Höfler, Gareth Ambler, Martina B. Goeldlin, Lena Obergottsberger, Gerit Wünsch, Markus Kneihsl, Wenpeng Zhang, Yang Du, Martina Locatelli, Hatice Ozkan, Philip S. Nash, Oliver Nistl, Larysa Panteleienko, Rom Mendel, Kitti Thiankhaw, Robert J. Simister, Hans Rolf Jäger, Christian Enzinger, David J. Seiffge, Thomas Gattringer, David J. Werring

**Affiliations:** From the Department of Neurology (S.F.-H., L.O., M.K., C.E., T.G.), Medical University of Graz, Austria; Department of Statistical Science (G.A.), University College London, United Kingdom; Department of Neurology (M.B.G., D.J.S.), University Hospital Bern, Inselspital, University of Bern, Switzerland; Institute for Medical Informatics, Statistics and Documentation (G.W.), Medical University of Graz, Austria; Stroke Research Centre (S.F.-H., W.Z., Y.D., M.L., H.O., P.S.N., L.P., R.M., K.T., R.J.S., D.J.W.), Department of Brain Repair & Rehabilitation, UCL Queen Square Institute of Neurology, United Kingdom; Division of Neuroradiology, Vascular and Interventional Radiology (O.N.), Department of Radiology, Medical University of Graz, Austria; and Neuroradiological Academic Unit (H.R.J.), Department of Brain Repair & Rehabilitation, UCL Queen Square Institute of Neurology, United Kingdom.

## Abstract

**Background and Objectives:**

Although most spontaneous intracerebral hemorrhages (ICHs) are due to cerebral small vessel diseases (SVDs), between 1 in 7 and 1 in 10 are due to a macrovascular cause. Rapid diagnosis has important therapeutic and prognostic implications but sometimes requires digital subtraction angiography (DSA), an invasive procedure which cannot be performed in all patients. MRI provides optimal sensitivity for markers of SVD but was not included in previous risk stratification scores. We aimed to create and validate a risk stratification score for macrovascular causes of ICH including MRI findings to guide diagnosis and further investigations.

**Methods:**

We pooled data from 2 large observational study cohorts (London/United Kingdom and Graz/Austria) of consecutive patients with ICH who had brain MRI and at least 1 angiographic modality within 90 days of symptom onset. The primary outcome was a macrovascular cause of ICH (arteriovenous malformation/dural arteriovenous fistula, aneurysm, cavernoma, or cerebral venous thrombosis), with the diagnosis based on neurovascular multidisciplinary meetings. Using lasso logistic regression, we built the MRI Assessment of the Causes of intRacerebral haemOrrhage (MACRO) score to assess the probability of a macrovascular cause. We performed internal validation using bootstrapping and external validation in an independent cohort (Bern/Switzerland).

**Results:**

We included 1,043 patients with ICH (mean age 66 years, 42% female), 78 of whom had a macrovascular cause (7.5%). The final score includes age (0–39, 40–69, or ≥70), location of ICH (lobar, deep, or infratentorial), and SVD markers on MRI (≥1 microbleed, ≥1 lacune, presence of cortical superficial siderosis, or white matter hyperintensities using the Fazekas scale). The MACRO score showed an optimism-adjusted *c*-statistic of 0.90 (95% CI 0.88–0.93), superior to existing CT-based scores (*p* < 0.001). In external validation, the *c*-statistic was 0.87 (95% CI 0.80–0.94). MACRO scores ≥6 (59.5% of patients) indicated a very low risk of a macrovascular cause (0.2%), while scores ≤2 (9% of patients) indicated a high risk (48.9%).

**Discussion:**

The MRI-based MACRO score shows excellent performance in predicting the likelihood of macrovascular causes of spontaneous intracerebral hemorrhage, making it useful in guiding further investigations. Important limitations include the observational study design and the performance of DSA in a minority of patients.

## Background

Spontaneous (nontraumatic) intracerebral hemorrhage (ICH) is a severe stroke subtype, accounting for 10%–30% of all strokes but half of the disability-adjusted life years lost due to stroke globally.^1^ The most frequent causes of ICH are sporadic cerebral small vessel diseases (SVDs; arteriolosclerosis or cerebral amyloid angiopathy)—reported to account for around 80% of cases^2^—and macrovascular causes (e.g., arteriovenous malformation, aneurysm, or cavernoma), which account for most of the remainder. Establishing the etiology of ICH influences treatment and assessment of prognosis, and is essential to guide rational clinical management to reduce the risk of recurrence. For example, some macrovascular causes are associated with potential rebleeding which may be prevented by specific treatment (surgery, endovascular, or medical treatment)—sometimes soon after ICH—necessitating their rapid and accurate identification.^3^

CT angiography (CTA) is a first-line investigation to identify most macrovascular causes of acute ICH; although earlier retrospective studies reported a very high sensitivity and specificity of CTA,^4,5^ a more recent prospective multicenter study found limited sensitivity of 74% when compared with the final diagnosis following a standardized investigation pathway.^6^ The reference standard for detection of most macrovascular causes is digital subtraction angiography (DSA), an invasive procedure requiring interventional neuroradiologist expertise and with a risk of complications, including access site hematoma and cerebral ischemia; the risk of complications in patients with ICH is reported to be 3%–5%.^7,8^

Therefore, it is very important to select patients with ICH for DSA according to the likely risk of finding a macrovascular cause; clinical-radiological prediction scores could support decision-making but are not used routinely. Previously proposed scores included clinical characteristics and CT findings,^9^ but cerebral SVD is more sensitively and comprehensively identified on MRI.^10,11^

Aside from supporting rapid diagnosis, triage for transfer to tertiary centers, and selection of patients for DSA, predicting the likelihood of a macrovascular cause for ICH may also help determine whether repeat MR imaging should be performed after resorption of the acute hematoma.

We aimed to develop and validate a new simple and practical risk score, termed MRI Assessment of the Causes of intRacerebral haemOrrhage (MACRO), to predict the likelihood of a macrovascular cause of ICH by incorporating MRI findings, demographic, and clinical data.

## Methods

### Study Population

We included data from 2 large observational cohort studies: the Stroke Investigation in North and Central London (SIGNAL) prospective registry of consecutive patients admitted to the Comprehensive Stroke Service based at the National Hospital of Neurology and Neurosurgery, University College London Hospitals NHS Foundation Trust, between January 2015 and October 2021, and a retrospective cohort consisting of patients with ICH admitted to the University Hospital of Graz, Austria, between 2008 and 2021. Details of both studies have been reported previously.^12-14^

**Figure 1:**
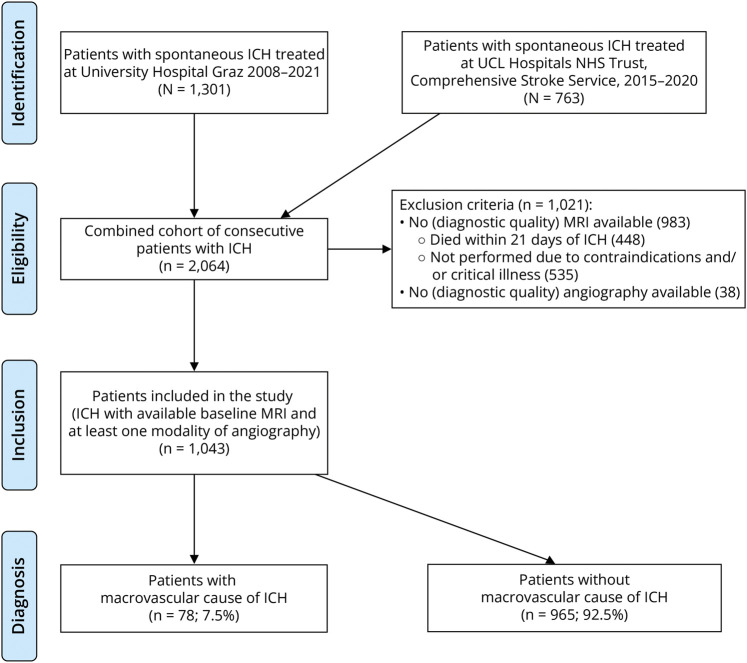
Study Flowchart (Derivation Cohort) ICH = intracerebral hemorrhage.

We included patients with ICH who had diagnostic quality MRI performed within 90 days of hospital admission with acute ICH and at least 1 modality of angiography (CTA, MR angiography, or DSA). A study flowchart is shown in Figure 1. Both institutions used MRI and at least 1 modality of CT or MR angiography in all patients with spontaneous ICH as a standard of care in the inpatient investigation to determine the cause during the entire study period, except for those with contraindications, severe disability or who declined the investigation. CT or MR venography was performed if there was any clinical suspicion for cerebral venous thrombosis. At both centers, a weekly neurovascular multidisciplinary meeting, consisting of vascular neurologists, neuroradiologists, and neurosurgeons, was used to determine etiology by consensus, discuss uncertain or “cryptogenic” cases, and plan further diagnostic evaluations, including DSA and repeat MRI. Follow-up was performed using all available electronic health records for both centers and surrounding hospital care systems and included assessment of recurrent ICH during follow-up as a potential sign of a missed macrovascular cause.^15^ In the case of cryptogenic ICH, diagnostic workup was repeated as determined by multidisciplinary consensus.

Macrovascular causes were classified as arteriovenous malformations, dural arteriovenous fistulas, aneurysms, cavernomas, and cerebral venous thromboses as previously reported.^9,16^ Hypertension was defined if any of the following criteria were fulfilled: a previously documented diagnosis of hypertension, ongoing antihypertensive therapy before ICH, or initiation of oral antihypertensive therapy during the hospital stay that was ongoing at discharge.

For inclusion, MRI protocols had to include as a minimum 1 structural (T2 or T2-weighted fluid attenuated inversion recovery) sequence and 1 blood (paramagnetic)-sensitive sequence (i.e., T2*-weighted gradient echo or susceptibility-weighted imaging), but usually included T2, T2-weighted fluid attenuated inversion recovery, T1, susceptibility-weighted imaging, and diffusion-weighted images (DWIs). We did not require contrast-enhanced MRI sequences for study inclusion. The first available MRI scan of sufficient quality was rated. MRI assessment for markers of cerebral SVD was performed by a neurovascular specialist (S.F.-H.) with supervision by 2 senior neurovascular specialists (T.G., C.E.) for the Graz cohort and in the SIGNAL cohort by 4 neurovascular specialists or clinical fellows (S.F.-H., Y.U., W.Z., M.L.) with resolution of discrepancies by a senior neuroradiologist (H.R.J.) and a neurovascular specialist (D.J.W.). MRI assessment of SVD markers^17^ included white matter hyperintensities according to the simplified Fazekas scale^18,19^; enlarged perivascular spaces according to a validated 4-point scale^20^; presence and number of lacunes; presence, number, and anatomical location of microbleeds according to the Microbleed Anatomical Rating Scale^21^; and presence and distribution of cortical superficial siderosis. We tested both continuous and dichotomized versions of these markers for score creation; dichotomization included moderate-to-severe white matter hyperintensities (Fazekas scale 2–3), ≥1 microbleed, and ≥1 lacune. In addition, we assessed acute lesions on DWI, intraventricular, and subarachnoid extension of ICH. Hematoma location was assessed according to the Cerebral Haemorrhage Anatomical RaTing inStrument^22^ and defined as lobar, supratentorial deep, brainstem, or cerebellar. We assessed clinical-based and CT-based characteristics included in previously proposed scores (the DIAGRAM score,^9^ the Secondary Intracerebral Hemorrhage Score,^23^ and the simple ICH score)^24^ to determine the risk of macrovascular causes.

An independent external validation cohort included consecutive patients with ICH, MRI, and noninvasive angiography or DSA admitted to the Inselspital Bern (Switzerland) during the period of January 2018 to December 2019. For this cohort, patient data were collected prospectively. Neuroimaging rating was performed based on the same guidelines as above by a neurovascular research fellow (M.G.) and supervised by a neurovascular specialist (D.S.). In this center, MRI (rather than CT) is the primary imaging modality used for acute stroke syndromes, if patient status allows for it. Therefore, rating for CT-based scores was not available in the validation cohort because many patients in the validation cohort did not have CT imaging.

### Statistical Analysis

We performed statistical analysis using STATA, version 18 (StataCorp LLC, College Station, TX). As a first step in model creation, we screened for clinical and radiologic variables using univariable analysis (categorical variables were compared by the Pearson χ^2^ test, normally distributed continuous variables by the unpaired Student *t* test, and nonnormal distributions using the Mann-Whitney *U* test) and included those with a *p*-value of <0.20 for further analysis. This cutoff was chosen to screen variables with potential contribution to the model but also exclude variables unlikely to show much effect. We included the following potential predictor variables: age, sex, hypertension, diabetes, anticoagulation, ICH location, presence of DWI-positive lesions, presence of intraventricular or subarachnoid hemorrhage, presence of microbleeds (with various cutoff options), presence of lacunes, severely enlarged perivascular spaces, white matter hyperintensities (both as a numerical variable and dichotomized to severe vs not), and presence of cortical superficial siderosis. Those variables were considered as they were (1) known risk factors of ICH in general, (2) related to macrovascular causes of ICH, or (3) related to SVD-related ICH.^9,14,23^ We accounted for missing data using multiple imputation with chained equations (10 imputations, all potential predictor and outcome variables included in the model). We then fitted a logistic regression model using lasso estimation (to guard against overfitting) to create an initial model to determine relationships between potential predictor variables with the target variable, that is, macrovascular ICH etiology. For the purpose of simplification without loss of clinically meaningful contribution, we calculated the individual contribution of variables to the model using a stepdown routine,^25^ excluded those variables with negligible contribution (contribution to total *r*^2^ of <1%) and refitted the prediction model using lasso regression analysis. We then rounded regression coefficients to produce integer scores for ease of use in clinical practice. We calculated the area under the curve (AUC) and performed bootstrapping mirroring our model's development in all steps to internally validate our score and obtain a final optimism-adjusted AUC.^26^ We used receiver-operator curve analysis to determine cutoff points of high sensitivity and high specificity. We performed a sensitivity analysis including only patients who had MRI within 2 weeks of ICH onset. We further compared our score by using likelihood ratio tests with previously proposed CT-based risk scores for macrovascular causes of ICH.

This article follows the transparent reporting of a multivariable prediction model for individual prognosis or diagnosis reporting guideline.^27^

### Standard Protocol Approvals, Registrations, and Patient Consents

The study was approved by the ethics committee of the Medical University of Graz (approval number 32-265 ex 19/20), as a Service Evaluation at the National Hospital of Neurology and Neurology (approval number 07-202324-SE) and by the ethical board of Bern (ID 2019-00689). As a retrospective cohort study including routinely collected data, the need for individual informed consent was waived.

### Data Availability

The data sets of the derivation cohorts are available from the corresponding author on reasonable request.

## Results

Of 2,064 consecutive patients with ICH, 983 were excluded because of missing MRI (448 died within the first 21 days of admission, and 535 did not have MRI, mostly due to critical illness or contraindications). Thirty-eight patients did not have any form of acute noninvasive angiography (Figure 1). We included 1,043 patients (mean age 65.8 ± 14.7 years, 57.8% male, Table 1). Arterial hypertension was the most frequent risk factor (745 patients, 71.4%). ICH location was lobar in 44.2%, supratentorial deep in 43.5%, and infratentorial (including brainstem and cerebellum) in 12.3% of participants. The median ICH volume was 9 mL (interquartile range [IQR] 3–23). Table 1 summarizes all of the other potential predictors evaluated for inclusion in the final model. Seventy-eight patients (7.3%) had a macrovascular cause of ICH (arteriovenous malformation or dural arteriovenous fistula in 33, cavernoma in 33, cerebral venous thrombosis in 10, and an aneurysm in 2). A detailed summary of ICH causes and modalities of detection is reported in eTables 1 and 2.

**Table 1:** Clinical Characteristics of Participants, Subdivided According to the Presence of a Macrovascular Cause of ICH in the Derivation Cohort

	All participants (n = 1,043)	Macrovascular cause (n = 78, 7.5%)	Other non-macrovascular etiologies (n = 965, 92.5%)	*p* Value
Clinical data				
Age, y, mean ± SD	65.8 ± 14.7	50.5 ± 17.4	67.0 ± 13.7	<0.001
Male sex, n (%)	603 (57.8)	46 (59.0)	557 (57.7)	0.83
Arterial hypertension, n (%)	745 (71.4)	32 (42.0)	713 (73.9)	<0.001
Diabetes mellitus, n (%)	180 (17.3)	9 (11.5)	171 (17.7)	0.17
Anticoagulation at time of index ICH, n (%)^a^	114 (13.6)	8 (12.5)	106 (13.7)	0.79
Hematoma location, n (%)				
Lobar	461 (44.2)	44 (56.4)	417 (43.2)	0.02
Supratentorial deep	454 (43.5)	6 (7.7)	448 (46.4)	<0.001
Infratentorial	128 (12.3)	28 (35.9)	100 (10.4)	<0.001
MRI findings				
Concomitant intraventricular hemorrhage, n (%)	279 (26.8)	18 (23.1)	281 (27.1)	0.44
Concomitant subarachnoid hemorrhage, n (%)	218 (21.0)	19 (24.4)	199 (20.8)	0.45
Cortical superficial siderosis, n (%)	121 (11.6)	1 (1.3)	120 (12.4)	0.003
Microbleeds, any, n (%)	635 (60.9)	15 (19.2)	620 (64.2)	<0.001
Microbleed number, median (IQR)	1 (0–7)	0 (0–0)	2 (0–8)	0.05
Lobar microbleed number, median (IQR)	0 (0–3)	0 (0–0)	0 (0–4)	<0.001
Deep microbleed number, median (IQR)	0 (0–2)	0 (0–0)	0 (0–2)	0.08
White matter hyperintensities, n (%)				<0.001
Simplified Fazekas scale 0	153 (14.7)	50 (64.1)	103 (10.7)	
Simplified Fazekas scale 1	308 (29.5)	18 (23.1)	290 (30.0)	
Simplified Fazekas scale 2	293 (28.1)	9 (11.5)	284 (29.4)	
Simplified Fazekas scale 3	289 (27.7)	1 (1.3)	288 (29.8)	
Lacunes, any, n (%)	320 (30.7)	3 (3.8)	317 (32.8)	<0.001
Severely ePVS (centrum semiovale)^b^	336 (36.4)	11 (15.3)	325 (38.2)	<0.001
Severely ePVS (basal ganglia)^c^	218 (23.1)	3 (4.2)	215 (24.7)	<0.001
Diffusion-weighted imaging positive lesions^d^	164 (16.1)	5 (6.4)	159 (16.9)	0.02

Abbreviations: ePVS = enlarged perivascular space; ICH = intracerebral hemorrhage; IQR = interquartile range.

a Data missing in 19.6% of patients.

b Missing in 11.5% of patients.

c Missing in 9.5% of patients.

d Missing in 2.1% of patients.

The median time from ICH to MRI was 3 days (IQR 1–8). Repeat MRI (at least 4 weeks after ICH onset) was performed in 36.8% of patients and DSA in 10.5% of patients. Dynamic (4-dimensional time-resolved) 3T MR angiography was performed as an alternative modality in 3.8% of patients who did not have DSA. Follow-up regarding recurrent ICH was available over a period of at least 6 months in 79.1% of patients, over at least 1 year in 73.9% of patients, and over at least 3 years in 50.5% (overall follow-up 4,819 patient-years, range 0–15 years).

White matter hyperintensities were present in a large proportion of the cohort (Fazekas score grade 0: 14.7%; grade 1: 29.5%; grade 2: 28.1%; and grade 3: 27.7%), as were cerebral microbleeds (≥1 in 60.9% of patients). Lacunes and enlarged perivascular spaces were found in a third to a quarter of patients, and cortical superficial siderosis in 11.6%. All markers of SVD were more frequently found in patients without macrovascular causes of ICH (all *p* < 0.01, Table 1).

### MACRO Score Creation

**Figure 2:**
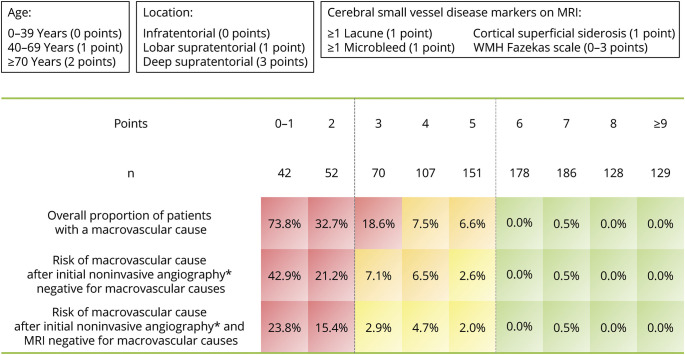
MRI to Assess ICH Etiology (MACRO) Score Observed risk categories are color-coded as <1% (green), 1%–5% (yellow), 5%–10% (orange), and >10% (red). Higher MACRO scores indicate a lower risk of a macrovascular cause. The overall proportion of patients with a macrovascular cause per MACRO score is reported, as well as the observed risk of detecting a macrovascular cause after initial CT angiography and after both MRI and CT angiography showing no evidence of a macrovascular cause. *CT or MR angiography. ICH = intracerebral hemorrhage; MACRO = MRI Assessment of the Causes of intRacerebral haemOrrhage.

Following univariable analyses and variable selection, we identified 9 potential predictors of macrovascular cause of ICH in lasso multivariable analysis: age, hypertension, ICH location, white matter hyperintensity grade, presence of microbleeds, presence of lacunes, presence of cortical superficial siderosis, severe enlarged perivascular spaces, and DWI-positive lesions. Owing to their negligible contribution to the model, we omitted DWI-positive lesions, severe enlarged perivascular spaces, and hypertension from the final model. The final MACRO score therefore consists of age, ICH location, and 4 markers of SVD: white matter hyperintensity grade, microbleeds, lacunes, and cortical superficial siderosis (individual regression coefficients shown in eTable 3). For all of these variables, there were no missing or imputed data. The score is visualized in Figure 2, which depicts the overall proportion of patients with a macrovascular cause based on the MACRO score and the observed probabilities of detecting a macrovascular cause of ICH in patients where initial CTA was negative for a macrovascular cause, and in those where both initial CTA and MRI were negative for a macrovascular cause. Visual rating instructions for the MACRO score are available in eAppendix 1.

**Figure 3:**
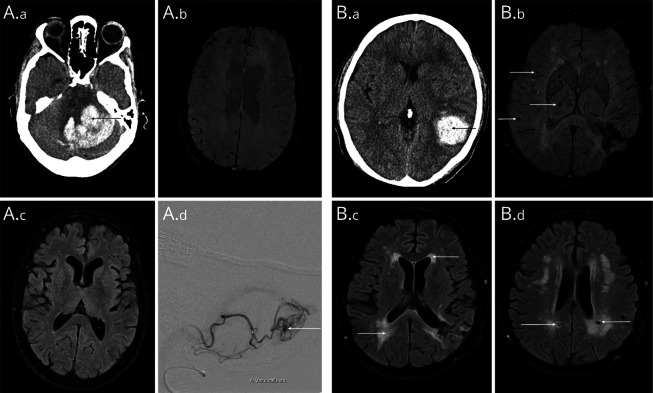
Neuroimaging Examples Showing Included Patients With Different Risks of a Macrovascular Cause (A) Patient in their fifties with cerebellar ICH (A.a). SWI and FLAIR sequences (A.b/A.c) showed no evidence of cerebral small vessel disease (MACRO score 1, indicating a high risk of a macrovascular cause). In dynamic MR angiography, a small AVM originating from the left posterior inferior cerebellar artery was suspected, which was confirmed on DSA (A.d). (B) Patient in their forties with a parietal lobar ICH (B.a). SWI shows several cerebral microbleeds in a mixed distribution (B.b), as well as confluent white matter hyperintensities and several lacunes (B.c/B.d). This patient had a MACRO score of 7, indicating a very low risk of a macrovascular cause. DSA and repeat MRI did not show any evidence of a macrovascular cause for the ICH. DSA = digital subtraction angiography; FLAIR = fluid-attenuated inversion recovery; ICH = intracerebral hemorrhage; MACRO = MRI Assessment of the Causes of intRacerebral haemOrrhage.

The discrimination performance of the score was excellent (optimism-adjusted AUC of 0.90, 95% CI 0.88–0.93). Calibration plots confirmed excellent calibration (calibration slope 1.03, 95% CI 0.81–1.25, eFigure 1). Figure 3 depicts neuroimaging examples and MACRO scores of 2 included patients. A MACRO score ≥6 (attained by 59.5% of the study population) indicated the likely *absence* of a macrovascular cause of ICH (0.2%; sensitivity 0.63, specificity 0.99, positive likelihood ratio 49.4; eTable 4), while a MACRO score ≤2 (8.9% of the study population) indicated the likely *presence* of a macrovascular cause (48.9%; sensitivity 0.59, specificity 0.95, positive likelihood ratio 11.9; eTable 5).

In a sensitivity analysis including only patients with MRI performed within 2 weeks of ICH (83.3%), the discrimination performance of the score was unchanged (optimism-adjusted AUC of 0.90, 95% CI 0.88–0.93).

### Comparison With Previously Proposed Scores

**Figure 4:**
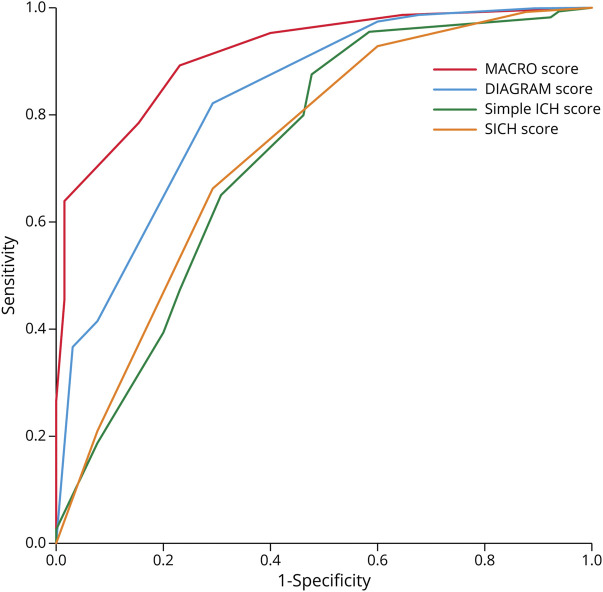
ROC Curves for Different Macrovascular Risk Scores in the Derivation Cohort of Patients With Spontaneous Intracerebral Haemorrhage ICH = intracerebral hemorrhage; MACRO = MRI Assessment of the Causes of intRacerebral haemOrrhage; ROC = receiver operating characteristic; SICH = spontaneous ICH.

We compared the performance of the MACRO score with 3 previously proposed risk scores. The DIAGRAM score showed good performance (*c*-statistic 0.83, 95% CI 0.78–0.88), while the Secondary Intracerebral Hemorrhage Score and the simple ICH score showed moderate performance (*c*-statistic 0.75, 95% CI 0.69–0.81 and 0.75, 95% CI 0.68–0.82, respectively). The MACRO score showed statistically significantly better discrimination than all of the other scores (*p* < 0.001 for all comparisons). Figure 4 depicts ROC curves for all compared scores.

### External Validation

We externally validated the MACRO score in a separate independent cohort of patients from a different center, consisting of 154 patients with ICH, available brain MRI and at least 1 modality of angiography (median age 67 years, 44% male); of these, 31.3% of patients had DSA and 33 (20.2%) had a final diagnosis of a macrovascular cause. This cohort is derived from the Bernese ICH registry, which included 375 patients with ICH in the study time frame, of whom 164 had MRI and 10 further patients were excluded because of missing noninvasive angiography (n = 8) or incomplete MRI (n = 2). More details are given in eTable 6. In this external cohort, the MACRO score also showed very good discrimination performance (*c*-statistic 0.87, 95% CI 0.80–0.94). In the validation cohort, the proposed cutoff of ≥6 for low risk of macrovascular causes (35.1% of patients in that cohort) also showed a very low proportion of macrovascular causes (3.7%), while patients within the cutoff ≤2 (implying a high risk of macrovascular causes) had a high proportion of macrovascular causes (15/29, 51.7%). A comparison of the clinical characteristics of the derivation cohort (delineated between study sites) and the validation cohort is given in eTable 7.

## Discussion

We describe the development and validation of the MACRO score, an MRI-based score to predict the risk of macrovascular causes in patients with spontaneous ICH, thus facilitating rational individualized diagnostic testing. The score is easily calculated, consisting of simple clinical-based and MRI-based variables available in daily clinical practice. Besides excellent discrimination performance, the score provides cutoff points with high predictive value for very low risk (0.2% with scores ≥6) and high risk (48.9% with scores ≤2) of a macrovascular cause of ICH. This information should be useful for rapid diagnosis (i.e., of cerebral SVD vs a macrovascular cause) and to guide clinical decision-making regarding the indication for further diagnostic workup for ICH (i.e., DSA, repeat MRI, or both).

In comparison with previous CT-based risk scores (the DIAGRAM score,^9^ Secondary Intracerebral Hemorrhage Score and simple ICH score), the MACRO score showed improved discrimination for the prediction of a macrovascular cause of ICH. We also found excellent agreement between the MACRO predicted and observed risks of a macrovascular cause. MRI allows for a more sensitive detection of a greater range of markers of cerebral SVD (including white matter hyperintensities, lacunes, microbleeds, or superficial siderosis) and of rarer causes of ICH including cavernomas and cortical vein thrombosis.^28^ Although we acknowledge that MRI is not yet standard of care in the diagnostic workup of ICH in many centers throughout the world, our data suggest that it can usefully improve the diagnostic pathway for ICH. Given the critical importance of determining the cause of ICH and the potential cost of unnecessary invasive testing with DSA (including specialist neuroradiology resources and the risks of complications), our results suggest that MRI should be considered for the investigation of ICH where available resources allow for it. The neuroimaging markers (cerebral microbleeds, lacunes, and cortical superficial siderosis), included in the MACRO score, are all part of the routine clinical assessment of brain MRI, and the simplified Fazekas score for white matter hyperintensities has previously shown good interrater reliability.^29-31^

A rational approach to the etiologic investigation of ICH is therefore to perform CT for immediate diagnosis of ICH presence and location, followed by early CTA to initially evaluate for macrovascular causes (and CT or MR venography if there is any concern for cerebral venous thrombosis) and then MRI when the patient is sufficiently stable (within days to weeks). Based on those investigations and the MACRO score, an informed decision can then be made whether it is reasonable to perform DSA or repeat MRI (after 1–3 months) to investigate underlying macrovascular causes.

Our study addresses a clear knowledge gap and priority for research outlined in the 2022 American Heart Association Guideline for the Management of Patients With Spontaneous Intracerebral Hemorrhage,^3^ which emphasized the importance of identifying markers of both microvascular and macrovascular pathologies underlying ICH. A specific need was identified to incorporate markers of cerebral SVD (such as white matter hyperintensities, lacunes, microbleeds, or superficial siderosis) in the classification of patients into high-risk or low-risk categories for harboring underlying macrovascular lesions. We found that while all markers of SVD are, as expected, associated with a lower risk of a macrovascular cause of ICH, white matter hyperintensity severity showed the strongest relationship to this risk. Moreover, the MACRO score may also have implications regarding the necessity of performing CT or MR angiography in the investigation of ICH where MRI is available because a high MACRO score (≥6) indicates a very low risk of a macrovascular cause even without the use of noninvasive angiography.

Our study has some limitations, the most important of which is that the reference diagnostic standard for detecting a macrovascular cause (DSA) was not performed in all patients. However, both routine noninvasive angiography and discussion in expert neurovascular multidisciplinary meetings were part of standard of care, and a study with routine DSA in all participants is not likely to be ethical or feasible given the risks associated with this test. Although age may have been a significant factor in deciding whether to perform DSA, potentially leading to detection bias in younger patients, the long follow-up period makes missed macrovascular causes unlikely because most of these have a high ICH recurrence risk. In both the derivation and validation cohorts, a substantial number of patients were excluded due to missing MRI—mostly due to severe ICH—which likely led to a selection bias toward patients with somewhat milder ICH. Although our findings might not be generalizable toward patients with more severe ICH, our included population represents ‘real-world’ ICH survivors in whom questions about the underlying cause of ICH are most likely to be raised. The overall number of macrovascular lesions was modest, even with the large study cohort, but included a broad range of causes (vascular malformations, cavernomas, and cerebral venous sinus thrombosis), consistent with previous studies on this topic. Although we used a detailed bootstrapping routine to internally validate our findings and corrected for optimism in our final *c*-statistic, we cannot fully exclude bias toward the MACRO score in our validation cohort and in the comparisons with previously proposed CT-based risk scores.

Strengths of our study include the use of data from those 2 large, unselected cohorts with harmonized investigation pathways without the exclusion of patients at low risk of a macrovascular cause. Indeed, if we had applied the same inclusion criteria as the DIAGRAM study, we would have excluded 69.6% of patients, giving a much higher DSA rate of 23.7%.

The generalizability of the MACRO score is underlined by excellent performance in an external validation cohort from a third independent center (with frequent usage of DSA), but further external validation in other larger cohorts will be important to determine the clinical utility of the score in practice. Future clinical trials could also help establish whether an MRI-based strategy based on the MACRO score could improve predictions of ICH outcome or recurrence risk.

## Appendix Authors

**Table:**

Name	Location	Contribution
Simon Fandler-Höfler, MD, PhD	Department of Neurology, Medical University of Graz, Austria	Drafting/revision of the manuscript for content, including medical writing for content; major role in the acquisition of data; study concept or design; analysis or interpretation of data
Gareth Ambler, PhD	Department of Statistical Science, University College London, United Kingdom	Drafting/revision of the manuscript for content, including medical writing for content; analysis or interpretation of data
Martina B. Goeldlin, MD, PhD	Department of Neurology, University Hospital Bern, Inselspital, University of Bern, Switzerland	Drafting/revision of the manuscript for content, including medical writing for content; major role in the acquisition of data
Lena Obergottsberger, MD	Department of Neurology, Medical University of Graz, Austria	Drafting/revision of the manuscript for content, including medical writing for content; major role in the acquisition of data
Gerit Wünsch, PhD	Institute for Medical Informatics, Statistics and Documentation, Medical University of Graz, Austria	Drafting/revision of the manuscript for content, including medical writing for content; major role in the acquisition of data
Markus Kneihsl, MD, PhD	Department of Neurology, Medical University of Graz, Austria	Drafting/revision of the manuscript for content, including medical writing for content
Wenpeng Zhang, MSc	Stroke Research Centre, Department of Brain Repair & Rehabilitation, UCL Queen Square Institute of Neurology, United Kingdom	Drafting/revision of the manuscript for content, including medical writing for content; major role in the acquisition of data
Yang Du, MD	Stroke Research Centre, Department of Brain Repair & Rehabilitation, UCL Queen Square Institute of Neurology, United Kingdom	Drafting/revision of the manuscript for content, including medical writing for content; major role in the acquisition of data
Martina Locatelli, MD	Stroke Research Centre, Department of Brain Repair & Rehabilitation, UCL Queen Square Institute of Neurology, United Kingdom	Drafting/revision of the manuscript for content, including medical writing for content; major role in the acquisition of data
Hatice Ozkan, PhD	Stroke Research Centre, Department of Brain Repair & Rehabilitation, UCL Queen Square Institute of Neurology, United Kingdom	Drafting/revision of the manuscript for content, including medical writing for content; major role in the acquisition of data
Philip S. Nash, MSCI	Stroke Research Centre, Department of Brain Repair & Rehabilitation, UCL Queen Square Institute of Neurology, United Kingdom	Drafting/revision of the manuscript for content, including medical writing for content; major role in the acquisition of data
Oliver Nistl, MD	Division of Neuroradiology, Vascular and Interventional Radiology, Department of Radiology, Medical University of Graz, Austria	Drafting/revision of the manuscript for content, including medical writing for content; major role in the acquisition of data
Larysa Panteleienko, MD, PhD	Stroke Research Centre, Department of Brain Repair & Rehabilitation, UCL Queen Square Institute of Neurology, United Kingdom	Drafting/revision of the manuscript for content, including medical writing for content; major role in the acquisition of data
Rom Mendel, MD	Stroke Research Centre, Department of Brain Repair & Rehabilitation, UCL Queen Square Institute of Neurology, United Kingdom	Drafting/revision of the manuscript for content, including medical writing for content; major role in the acquisition of data
Kitti Thiankhaw, MD	Stroke Research Centre, Department of Brain Repair & Rehabilitation, UCL Queen Square Institute of Neurology, United Kingdom	Drafting/revision of the manuscript for content, including medical writing for content; major role in the acquisition of data
Robert J. Simister, PhD	Stroke Research Centre, Department of Brain Repair & Rehabilitation, UCL Queen Square Institute of Neurology, United Kingdom	Drafting/revision of the manuscript for content, including medical writing for content
Hans Rolf Jäger, MD	Neuroradiological Academic Unit, Department of Brain Repair & Rehabilitation, UCL Queen Square Institute of Neurology, United Kingdom	Drafting/revision of the manuscript for content, including medical writing for content; major role in the acquisition of data
Christian Enzinger, MD	Department of Neurology, Medical University of Graz, Austria	Drafting/revision of the manuscript for content, including medical writing for content
David J. Seiffge, MD	Department of Neurology, University Hospital Bern, Inselspital, University of Bern, Switzerland	Drafting/revision of the manuscript for content, including medical writing for content; major role in the acquisition of data
Thomas Gattringer, MD, PhD	Department of Neurology, Medical University of Graz, Austria	Drafting/revision of the manuscript for content, including medical writing for content; major role in the acquisition of data; study concept or design
David J. Werring, FRCP, PhD	Stroke Research Centre, Department of Brain Repair & Rehabilitation, UCL Queen Square Institute of Neurology, United Kingdom	Drafting/revision of the manuscript for content, including medical writing for content; major role in the acquisition of data; study concept or design; analysis or interpretation of data

## Glossary

AUC: area under the curve
CTA: CT angiography
DSA: digital subtraction angiography
DWI: diffusion-weighted imaging
ICH: intracerebral hemorrhage
IQR: interquartile range
MACRO: MRI Assessment of the Causes of intRacerebral haemOrrhage
SIGNAL: Stroke Investigation in North and Central London
SVD: small vessel disease
